# Y chromosome of the inbred mouse KK/Ta strain is associated with reduced body size in Y-consomic strains

**DOI:** 10.1186/1756-0500-6-64

**Published:** 2013-02-18

**Authors:** Jun-ichi Suto

**Affiliations:** 1 Agrogenomics Research Center, National Institute of Agrobiological Sciences, Tsukuba, Ibaraki 305-8634, Japan

**Keywords:** *A*^*y*^ allele, Body length, Body weight, Body size, Consomic mice, *Dh*, Y chromosome

## Abstract

**Background:**

We have established 17 Y chromosome consomic (Y-consomic) mouse strains in an inbred DH/Sgn strain. In this study, based on investigations in four different genetic backgrounds, we proved that the Y chromosome of the inbred mouse KK/Ta strain is associated with reduced body size.

**Findings:**

In the DH-Chr Y-+/+ background, Y chromosome substitution significantly decreased the body weight in DH-Chr Y^KK^-+/+ and DH-Chr Y^SJL^-+/+ strains, and the DH-Chr Y^KK^-+/+ strain was the lightest among the 17 Y-consomic strains. In the DH-Chr Y-*Dh*/+ background (*Dh*/+ mice have skeletal malformations and are usually lighter than +/+ mice), although Y chromosome substitution did not significantly alter the body weight, the DH-Chr Y^KK^-*Dh*/+ strain was the lightest among the 17 Y-consomic-*Dh*/+ strains. In the (B6.Cg-*A*^*y*^ × DH-Chr Y) F_1_-+/+ background, Y chromosome substitution significantly decreased the body weight and length in the (B6.Cg-*A*^*y*^ × DH-Chr Y^KK^) F_1_ hybrids. In the (B6.Cg-*A*^*y*^ × DH-Chr Y) F_1_-*A*^*y*^/+ background (*A*^*y*^ causes obesity and promotes linear growth), Y chromosome substitution significantly decreased body weight and length in the (B6.Cg-*A*^*y*^ × DH-Chr Y^KK^) F_1_-*A*^*y*^/+ hybrids.

**Conclusion:**

A body-size-reducing effect of the Y chromosome of the KK/Ta mouse strain was observed irrespective of genetic background. The effect was observed in the presence of *Dh* and *A*^*y*^, the autosomal dominant mutations, both of which are known to have substantial effects on body size. These results suggest that there are Y-linked genes that control the body size in mice.

## Findings

### Background

We have established 17 Y chromosome consomic (hereafter Y-consomic) mouse strains in an inbred DH/Sgn (hereafter DH) strain. There was a wide spectrum of variation in body weight and testis weight among the Y-consomic mouse strains [1,2]. Thus, it was expected that there were Y-linked genes associated with body weight and testis weight. We identified several SNPs and gene polymorphisms that were associated with testis weight variation when the trait was evaluated as a quantitative trait [2]. Although we have not yet identified SNPs and gene polymorphisms associated with body weight, we noted that the DH-Chr Y^KK^ strain was lighter than other Y-consomic strains [1]. Therefore, we further investigated the effect of the Y chromosome by incorporating additional mice in this study. Based on the investigation in four different genetic backgrounds, we proved that the Y chromosome of the inbred mouse KK/Ta strain is associated with reduced body weight and length.

First, we analyzed Y-consomic strains in the DH strain background. Because DH includes both +/+ and *Dh*/+ genotypes at the *dominant hemimelia* (*Dh*) locus on chromosome 1, each Y-consomic strain includes both +/+ and *Dh*/+ mice. Some skeletal elements are lost in *Dh*/+ mice; therefore, *Dh*/+ mice are usually lighter than +/+ littermates (see Methods for details). We next analyzed Y-consomic strains with the *Dh* mutation. We further investigated the Y-consomic strains in combination with *A*^*y*^, the obesity mutation. The *A*^*y*^ allele at the agouti locus on chromosome 2 is known to cause obesity and promote linear growth in mice (see Methods for details). When the males of each Y-consomic strain were crossed with females of the B6.Cg-*A*^*y*^ strain, the F_1_ generation consisted of *A*^*y*^ (yellow, *A*^*y*^/+) and non-*A*^*y*^ (agouti, +/+) mice. We analyzed the F_1_-+/+ and F_1_-*A*^*y*^ hybrids. This analysis allowed us to evaluate the effect of the Y chromosome in obese (F_1_*A*^*y*^) animals as well as in addition to non-obese (F_1_ non-*A*^*y*^) animals in the same genetic background. Thus, we investigated the effect of the Y chromosome in the presence of autosomal dominant mutations, both of which substantially affected body size.

### Methods

#### Mice

The following Y-consomic strains were used in this study: DH-Chr Y^A^ (Y chromosome from A/J strain), DH-Chr Y^AKR^ (AKR/J), DH-Chr Y^B6^ (C57BL/6J), DH-Chr Y^BALB^ (BALB/cA), DH-Chr Y^C3H^ (C3H/HeJ), DH-Chr Y^CAST^ (CAST/EiJ), DH-Chr Y^CBA^ (CBA/N), DH-Chr Y^CF1^ (CF1/Sgn), DH-Chr Y^DBA^ (DBA/2J), DH-Chr Y^DDD^ (DDD/Sgn), DH-Chr Y^DH^ (identical to DH), DH-Chr Y^KK^ (KK/Ta), DH-Chr Y^RF^ (RF/J), DH-Chr Y^RR^ (RR/Sgn), DH-Chr Y^SJL^ (SJL/J), DH-Chr Y^SS^ (SS/Sgn), and DH-Chr Y^SWR^ (SWR/J). B6.Cg-*A*^*y*^ strain was purchased from the Jackson Laboratory (Bar Harbor, ME, USA) and maintained at the National Institute of Agrobiological Sciences (NIAS, Tsukuba, Japan). Each Y-consomic strain included *Dh*/+ and +/+ mice with respect to the genotype at the *Dh* locus. *Dh* causes visceral and skeletal malformations of various degrees of severity [3,4]. Visceral abnormalities include a small stomach, short intestine, hydropic kidneys, and congenital absence of the spleen. Skeletal malformations appear in the trunk caudally from the thorax, particularly in the hindlimbs. The abnormalities induced by *Dh* are expressed more severely in *Dh*/*Dh* than in *Dh*/+ animals. Because *Dh*/*Dh* mice die shortly after birth owing to their visceral abnormalities, only heterozygous *Dh*/+ mice were available for this study. The skeletal malformations in *Dh*/+ mice are worth mentioning. In *Dh*/+ mice, the number of lumbar vertebrae is reduced to five, as opposed to six in +/+ mice. Loss of the hallux (i.e., presence of only four digits) is commonly observed in *Dh*/+ mice. However, triphalangy of the hallux (i.e., presence of five digits with an extra phalange on the hallux) is also commonly observed. Polydactyly is sometimes observed and is associated with an additional phalange on the hallux (the number of metatarsal bones do not exceed five even in the case of polydactyly). Although the fibula is rarely affected, various lengths of the distal part of the tibia are frequently lost. Thus, *Dh* is associated essentially with reduction of skeletal elements. *Dh*/+ mice were distinguished from +/+ mice by the presence of hindlimb malformation, and the *Dh*/+ genotype was confirmed by the absence of the spleen on laparotomy. The Y-consomic strains in a DH background are hereafter designated as DH-Chr Y-+/+ and DH-Chr Y-*Dh*/+ for convenience.

We also investigated (♀B6.Cg-*A*^*y*^ × ♂DH-Chr Y-+/+) F_1_ hybrids. The *A*^*y*^ allele at the agouti locus causes obesity and promotes linear growth in mice [5]. In normal mice, the agouti gene is expressed only in the skin [6,7], and it regulates pigmentation by serving as an inverse agonist of the melanocortin 1 receptor (MC1R) [8,9]. However, in *A*^*y*^ mice, the *A*^*y*^ allele is associated with a large deletion, causing agouti gene expression to be aberrantly controlled by the unrelated *Raly* gene promoter and leading to its ectopic overexpression [7,10-12]. As a result, *A*^*y*^ mice have a yellow coat color and develop maturity-onset obesity. The yellow coat makes it possible to visually distinguish *A*^*y*^ mice from non-*A*^*y*^ mice. Obesity in *A*^*y*^ mice is believed to be a consequence of the agouti proteins serving as a constitutive antagonist of the melanocortin 3 receptor (MC3R) and melanocortin 4 receptor (MC4R) by mimicking the action of the agouti-related protein [13-15]. Thus, *A*^*y*^ mice are heavier and longer than their non-*A*^*y*^ littermates. Importantly, mice homozygous for the *A*^*y*^ allele are embryonic lethal; therefore, living *A*^*y*^ mice are invariably heterozygotes. The F_1_ Y-consomic mice were designated as F_1_-+/+ and F_1_*A*^*y*^/+. Strain designations and numbers of mice used in this study are summarized in Table 1.

**Table 1:** Genetic backgrounds and numbers of mice in the Y-consomic strains used in this study

**Y-donor strain**	**DH-Chr Y-+/+**	**DH-Chr Y-*Dh*/+**	**(♀B6.Cg-*A***^***y***^ **× ♂DH-Chr Y-+/+) F**_**1**_**-+/+ (F**_**1**_**-+/+)**^**a**^	**(♀B6.Cg-*A***^***y***^ **× ♂DH-Chr Y-+/+) F**_**1**_**-*A***^***y***^**/+ (F**_**1**_**-*A***^***y***^**/+)**^**a**^
A/J (A)^a^	27	22	18	12
AKR/J (AKR)	37	34	5	13
C57BL/6J (B6)	32	24	17	18
BALB/cA (BALB)	24	30	7	13
C3H/HeJ (C3H)	40	24	8	15
CAST/EiJ (CAST)	26	21	12	12
CBA/N (CBA)	21	16	13	10
CF1/Sgn (CF1)	21	27	12	10
DBA/2J (DBA)	24	15	11	13
DDD/Sgn (DDD)	41	25	16 (15)^b^	13
DH/Sgn (DH)	19	40	9	10
KK/Ta (KK)	24	12	16	12
RF/J (RF)	32	16	8	10
RR/Sgn (RR)	26	12	13	11
SJL/J (SJL)	29	23	16	17
SS/Sgn (SS)	22	5	18	17
SWR/J (SWR)	27	20	10	12
Total	472	366	209 (208)^a^	218

^a^Abbreviation is shown in parentheses. ^b^We failed to determine the body length of one (♀B6.Cg-*A*^*y*^ × ♂DH-Chr Y^DDD^-+/+) F_1_-+/+ mouse.

All mice were maintained in a specific-pathogen-free facility with a regular light cycle (12 h light and 12 h dark) and controlled temperature (23 ± 1°C) and humidity (50%). Food and water were freely available throughout the experimental period. DH-Chr Y-+/+ and DH-Chr Y-*Dh*/+ strains were fed a CE-2 (CLEA Japan Inc., Tokyo) and F_1_-+/+ and F_1_-*A*^*y*^/+ hybrids were fed a CRF-1 (Oriental Yeast Co. Ltd., Tokyo). We are uncertain whether or not the difference in the lot of diet might have any impacts on body weights and/or body sizes of mice. All animal experiments were performed in accordance with guidelines approved by the Institutional Animal Care and Use Committee of NIAS.

#### Phenotyping

At the age of 80 days for DH-Chr Y-+/+ and DH-Chr Y-*Dh*/+ strains and at the age of 16 weeks for F_1_-+/+ and F_1_-*A*^*y*^/+ hybrids, the mice were weighed on an electric balance to the nearest 0.01 g after 4 h fasting. For F_1_-+/+ and F_1_-*A*^*y*^/+ hybrids, the anal–nasal length and tail length of each mouse were measured to the nearest 0.01 mm with digital calipers. Body length was defined as the anal–nasal length.

#### Statistics

Normality of distribution of the trait data for each Y-consomic strain was tested by the Shapiro–Wilk W test (JMP 8, SAS Institute Inc., Cary, NC, USA). If the trait values did not follow a normal distribution, they were normalized using the Box–Cox transformation.

Statistical comparison of two groups was performed by the Student’s t-test. Effects of Y chromosome substitution were assessed using Dunnett’s multiple-comparison tests with the background DH strain as a reference. P <0.05 was considered statistically significant.

### Results

**Figure 1:**
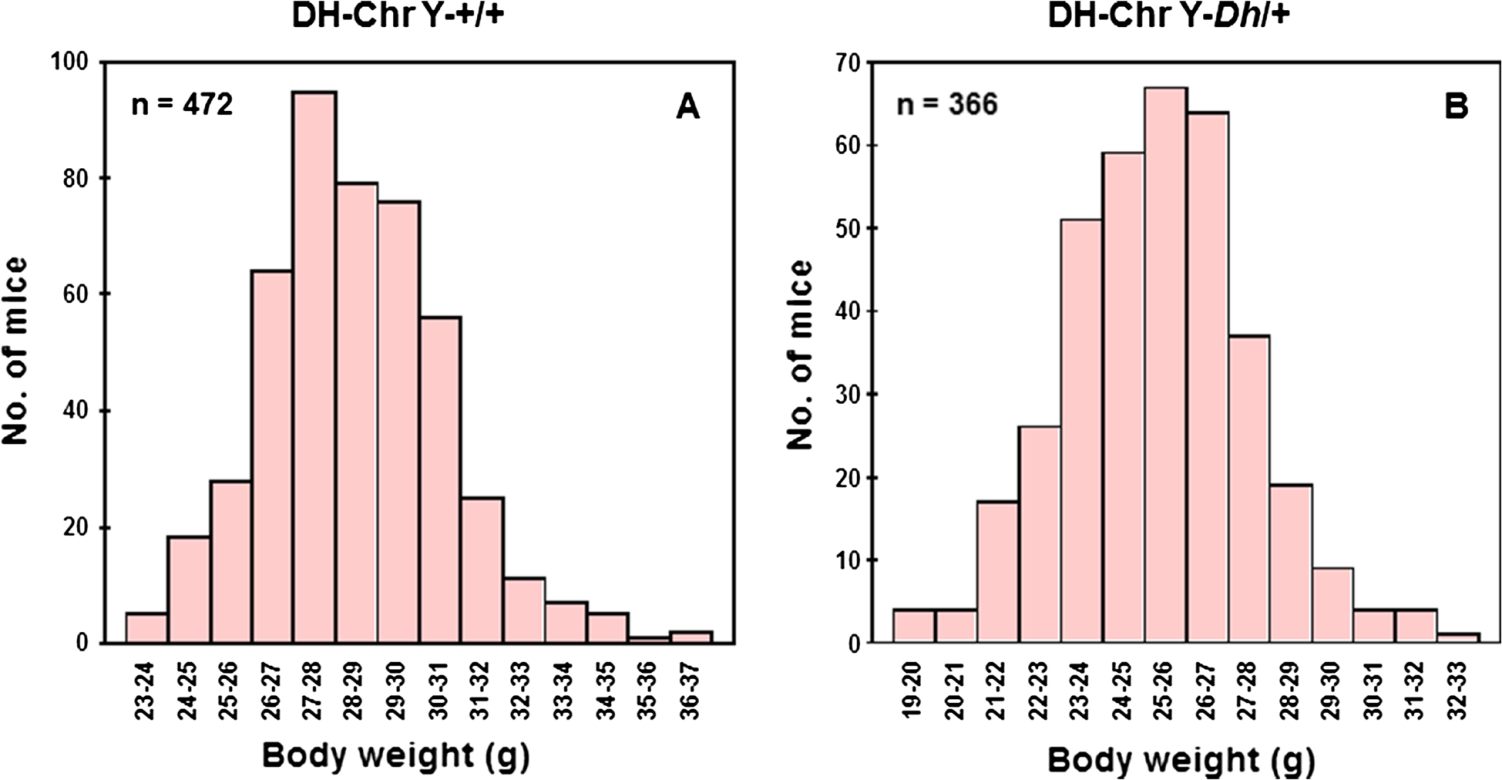
**The distribution of body weight in DH-Chr Y-+/+ (A) and DH-Chr Y-*Dh*/+ (B) strains.**

**Figure 2:**
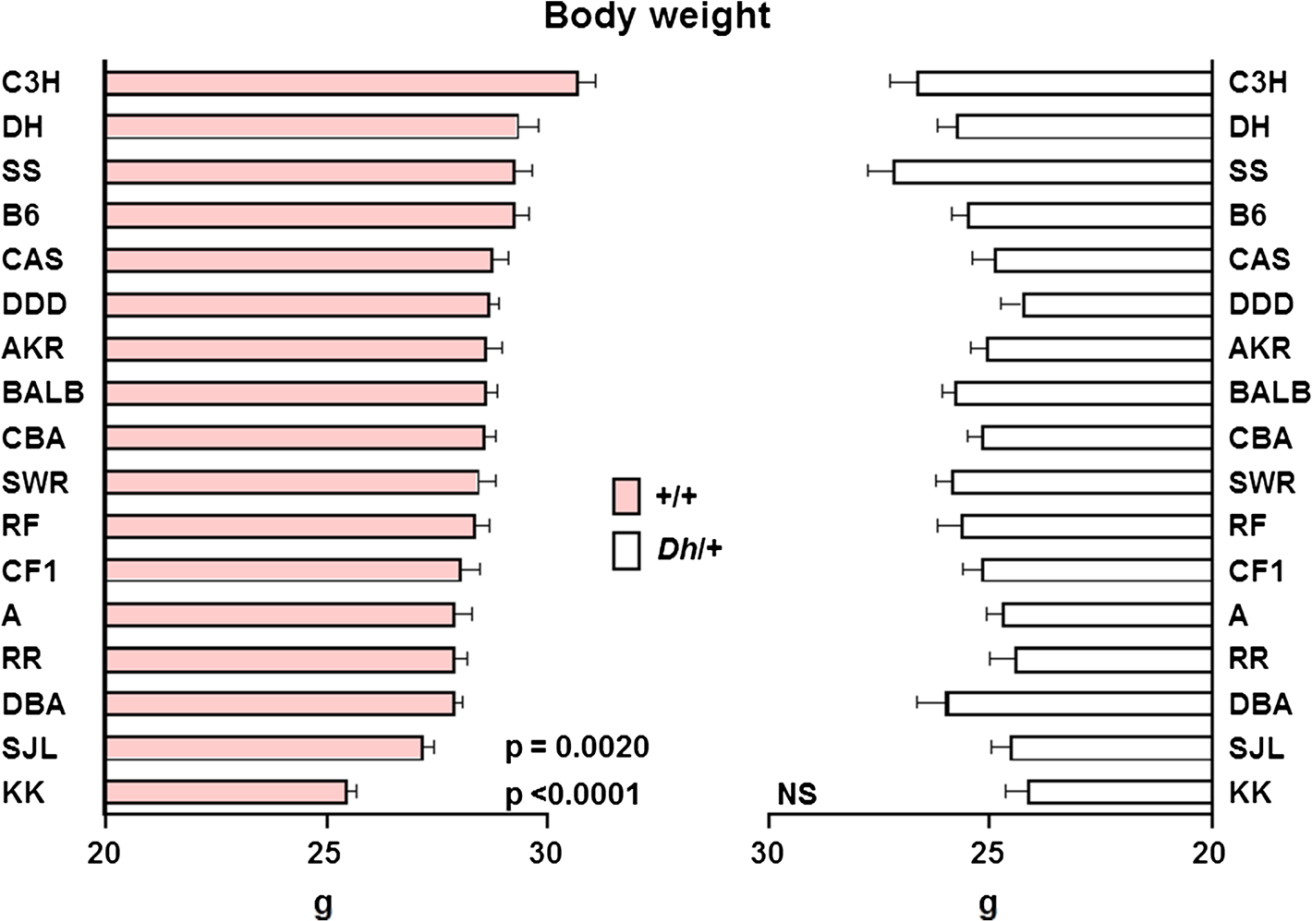
**The effect of Y chromosome substitution on body weight in DH-Chr Y-+/+ (left column) and DH-Chr Y-*Dh*/+ (right column) strains. **The difference between DH-Chr Y^DH^ strain and others were analyzed in both +/+ and *Dh*/+ backgrounds and only the difference between DH-Chr Y^DH^-+/+ and DH-Chr Y^KK^-+/+ strains and between DH-Chr Y^DH^-+/+ and DH-Chr Y^SJL^-+/+ strains was statistically significant. DH-Chr Y-+/+ strains are sorted in descending order from the top to the bottom.

Figure 1 shows the distributions of body weight in 472 DH-Chr Y-+/+ (A) and 366 DH-Chr Y-*Dh*/+ (B) strains. As expected, average body weight was significantly higher in +/+ strains (mean ± SE, 28.48 ± 0.10 g) than in *Dh*/+ strains (25.29 ± 0.12 g). Body weight showed bell-shaped distribution curves in both mice. Strictly, the distribution of body weight in DH-Chr Y-*Dh*/+ strains followed a normal distribution but that of DH-Chr Y-+/+ strains did not. Therefore, Box–Cox transformation was applied to the DH-Chr Y-+/+ strains before subsequent analyses. Figure 2 shows the effect of the Y chromosome substitution on body weight in DH-Chr Y-+/+ and DH-Chr Y-*Dh*/+ strains. In the DH-Chr Y-+/+ background, Y chromosome substitution significantly decreased body weight in DH-Chr Y^SJL^-+/+ and DH-Chr Y^KK^-+/+ strains. The DH-Chr Y^KK^-+/+ strain was the lightest among the DH-Chr Y-+/+ strains. In the DH-Chr Y-*Dh*/+ background, although Y chromosome substitution did not significantly alter the body weight, the DH-Chr Y^KK^-*Dh*/+ strain was the lightest among the strains. The difference between DH-Chr Y^DH^ and DH-Chr Y^C3H^ strains was not statistically significant in both +/+ and *Dh*/+ backgrounds.

**Figure 3:**
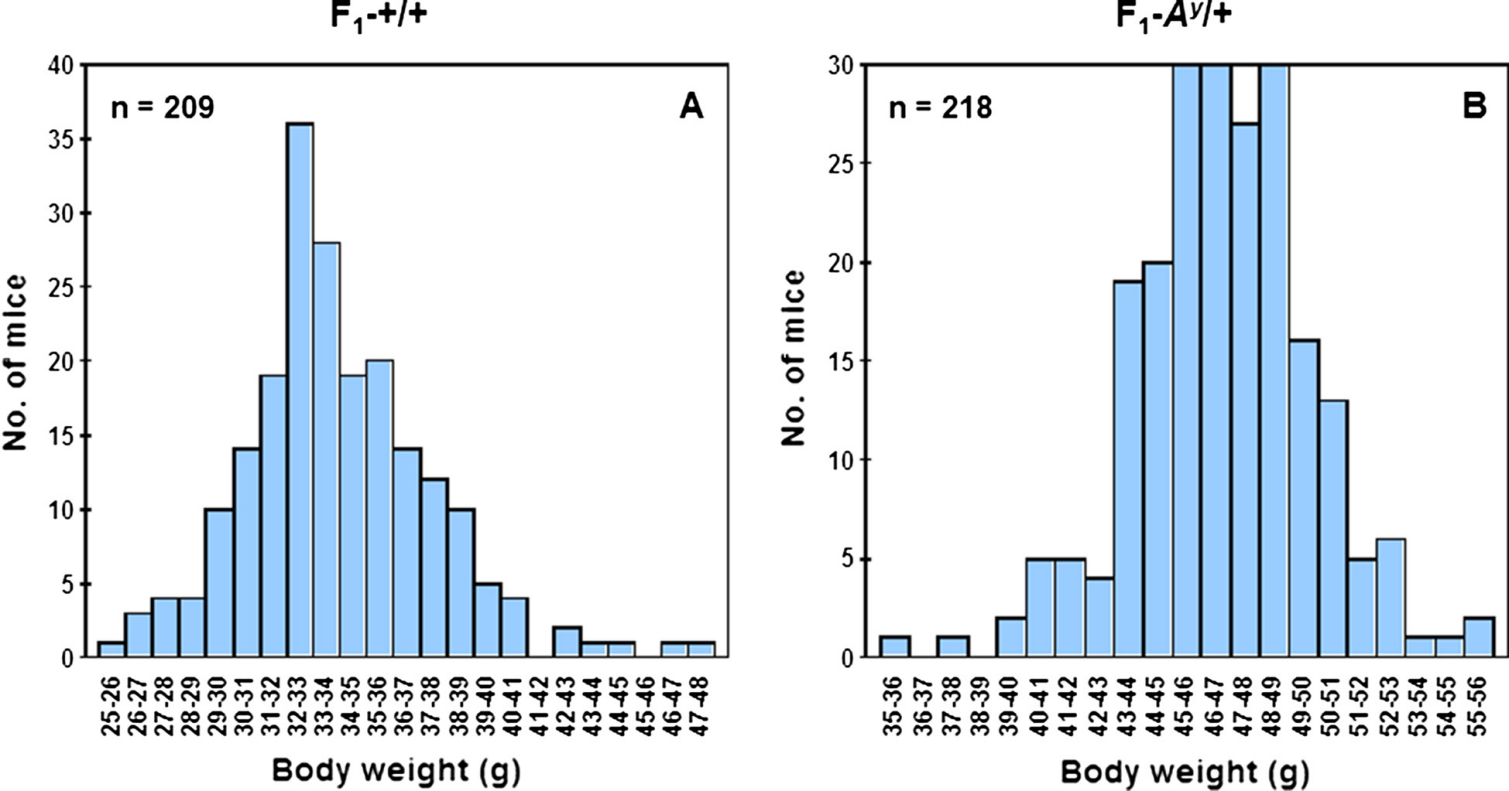
**The distribution of body weight in F**_**1**_**-+/+ (A) and F**_**1**_**-*A***^***y***^**/+ (B) hybrids.**

**Figure 4:**
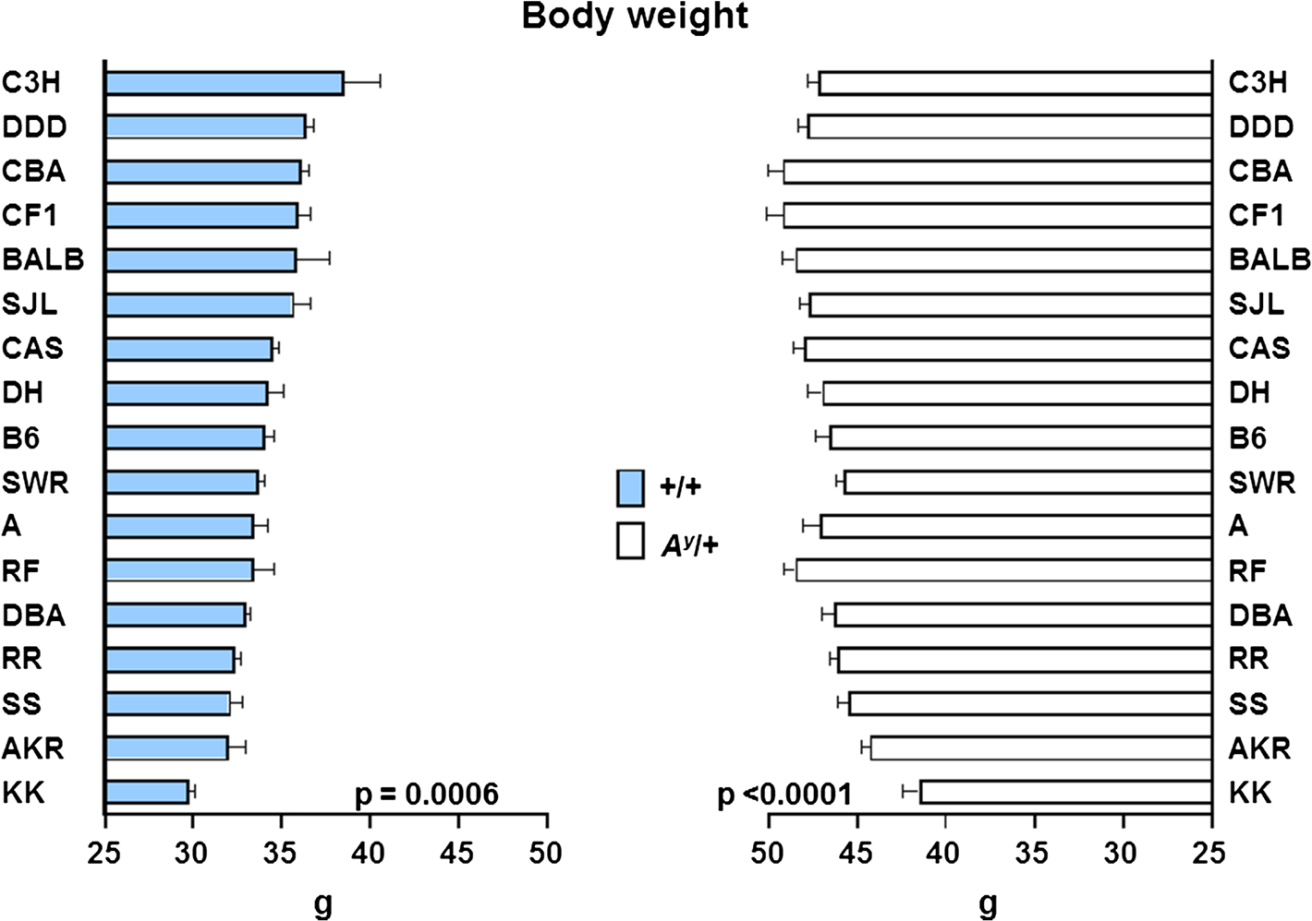
**The effect of Y chromosome substitution on body weight in F**_**1**_**-+/+ (left column) and F**_**1**_**-*A***^***y***^**/+ (right column) hybrids. **The difference between (B6.Cg-*A*^*y*^ × DH-Chr Y^DH^) F_1 _hybrids and others were analyzed and only the difference between (B6.Cg-*A*^*y*^ × DH-Chr Y^DH^) F_1 _and (B6.Cg-*A*^*y*^ × DH-Chr Y^KK^) F_1 _hybrids was statistically significant in both +/+ and *A*^*y*^/+ backgrounds. F_1_-+/+ hybrids are sorted in descending order from the top to the bottom.

Figure 3 shows the distributions of body weight in 209 F_1_-+/+ (A) and 218 F_1_-*A*^*y*^/+ (B) hybrids. As expected, average body weight was significantly higher in *A*^*y*^/+ mice (mean ± SE, 46.69 ± 0.21 g) than in +/+ mice (33.98 ± 0.24 g). The distribution of body weight in F_1_-*A*^*y*^/+ hybrids followed a normal distribution but that of F_1_-+/+ hybrids did not. Therefore, Box–Cox transformation was applied to the F_1_-+/+ hybrids before subsequent analyses. Figure 4 shows the effect of Y chromosome substitution on body weight in F_1_-+/+ and F_1_-*A*^*y*^/+ hybrids. In the F_1_-+/+ and F_1_-*A*^*y*^/+ backgrounds, Y chromosome substitution significantly decreased body weight in the (B6.Cg-*A*^*y*^ × DH-Chr Y^KK^) F_1_-+/+ and (B6.Cg-*A*^*y*^ × DH-Chr Y^KK^) F_1_-*A*^*y*^/+ hybrids, respectively. Although average body weight of the (B6.Cg-*A*^*y*^ × DH-Chr Y^C3H^) F_1_ hybrids was the highest in +/+ background, the difference between (B6.Cg-*A*^*y*^ × DH-Chr Y^DH^) F_1_-+/+ and (B6.Cg-*A*^*y*^ × DH-Chr Y^C3H^) F_1_-+/+ hybrids was not statistically significant.

**Figure 5:**
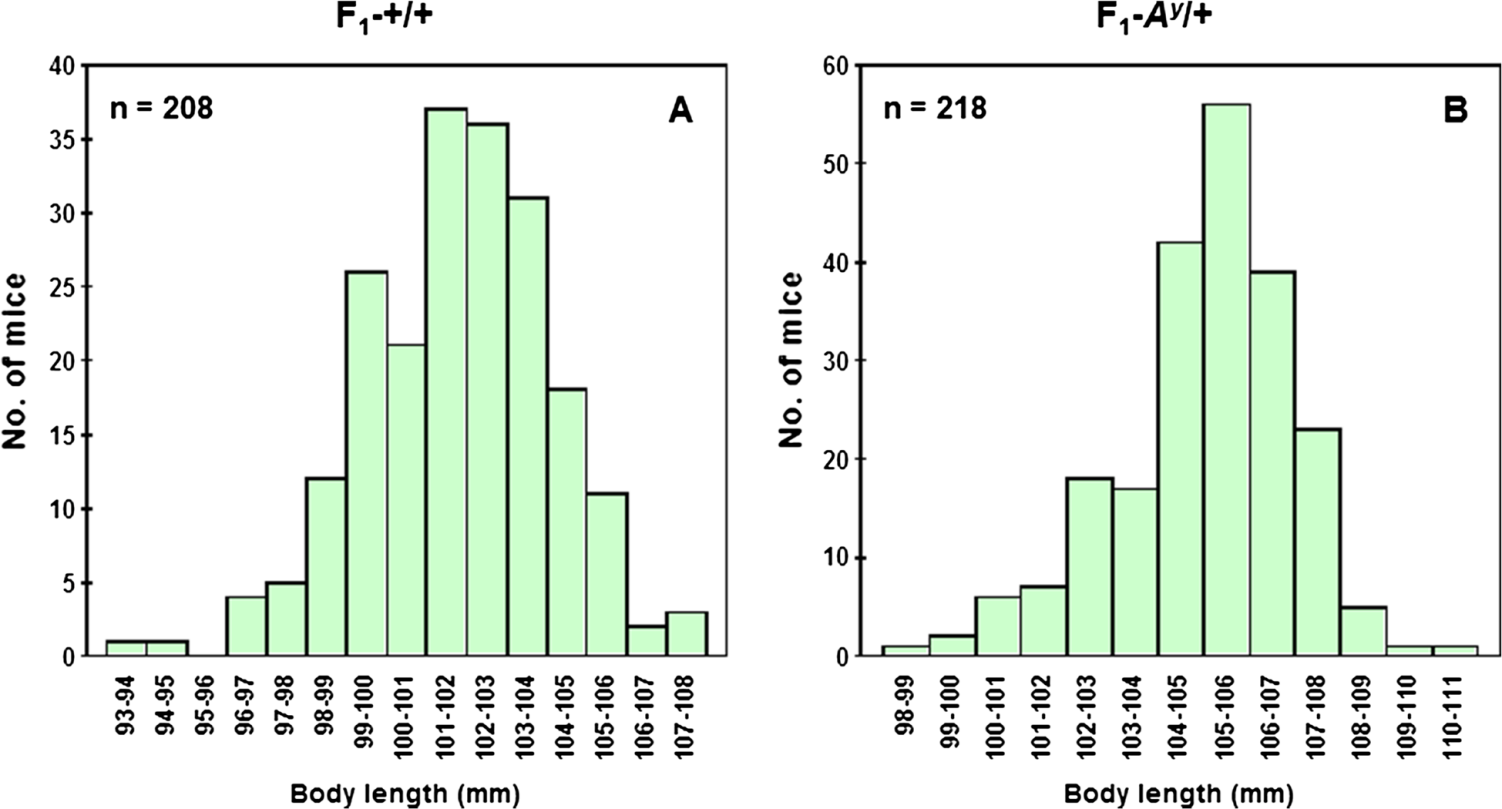
**The distribution of body length in F**_**1**_**-+/+ (A) and F**_**1**_**-*A***^***y***^**/+ (B) hybrids.**

**Figure 6:**
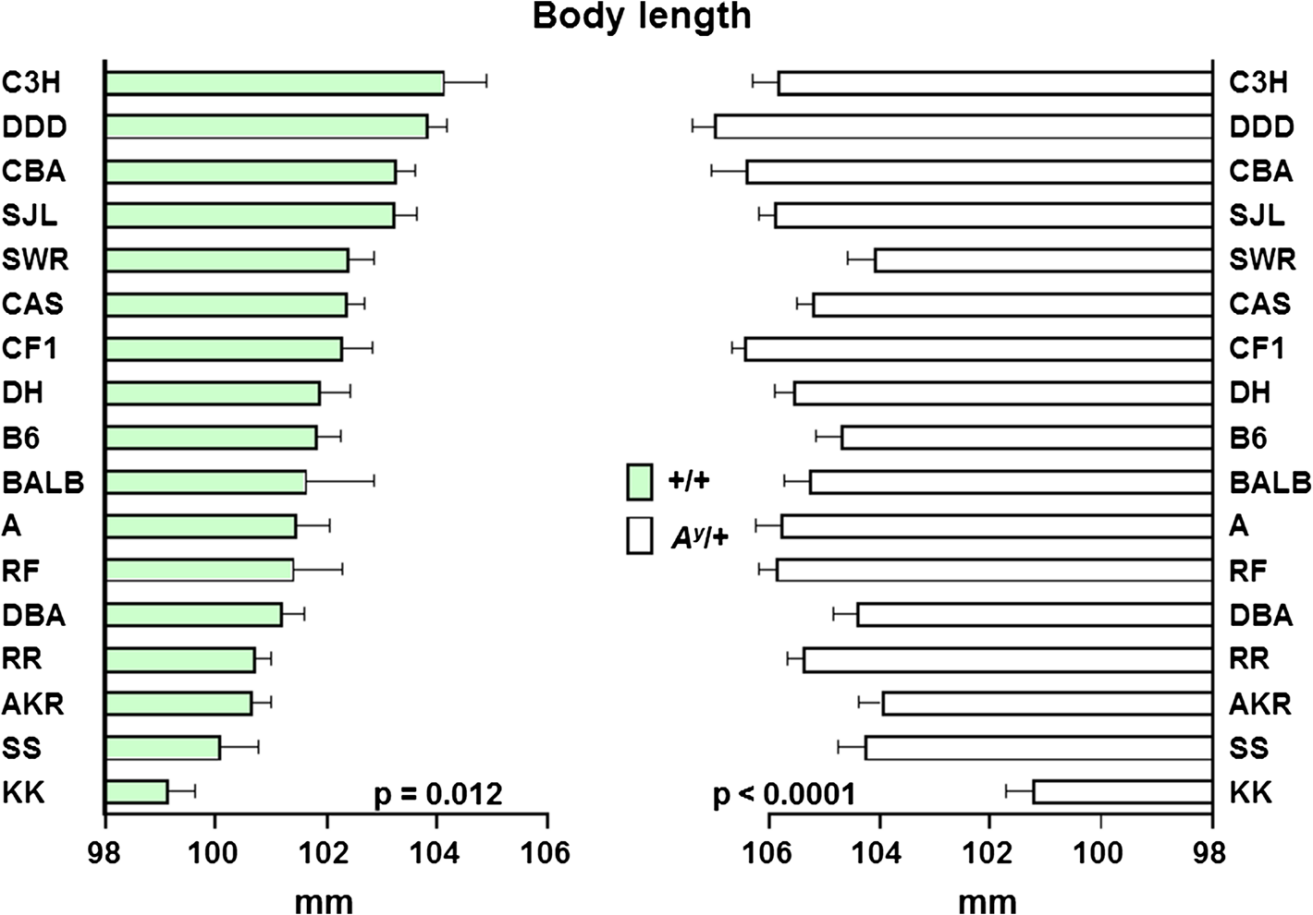
**The effect of Y chromosome substitution on body length in F**_**1**_**-+/+ (left column) and F**_**1**_**-*A***^***y***^**/+ (right column) hybrids. **The difference between (B6.Cg-*A*^*y*^ × DH-Chr Y^DH^) F_1 _hybrids and others were analyzed and only the difference between (B6.Cg-*A*^*y*^ × DH-Chr Y^DH^) F_1 _and (B6.Cg-*A*^*y*^ × DH-Chr Y^KK^) F_1 _hybrids was statistically significant in both +/+ and *A*^*y*^/+ backgrounds. F_1_-+/+ hybrids are sorted in descending order from the top to the bottom.

Figure 5 shows the distributions of body length in 208 F_1_-+/+ (A) and 218 F_1_-*A*^*y*^/+ (B) hybrids (body length was measured only in F_1_ strains). Average body length was significantly greater in *A*^*y*^/+ mice (mean ± SE, 105.08 ± 0.13 mm) than in +/+ mice (101.79 ± 0.16 mm). The distribution of body length in F_1_-+/+ hybrids followed a normal distribution but that of F_1_-*A*^*y*^/+ hybrids did not. Therefore, Box–Cox transformation was applied to the F_1_-*A*^*y*^/+ hybrids before subsequent analyses. Figure 6 shows the effect of the Y chromosome substitution on body length in F_1_-+/+ and F_1_-*A*^*y*^/+ hybrids. In the F_1_-+/+ and F_1_-*A*^*y*^/+ backgrounds, Y chromosome substitution significantly decreased body length in the (B6.Cg-*A*^*y*^ × DH-Chr Y^KK^) F_1_-+/+ and (B6.Cg-*A*^*y*^ × DH-Chr Y^KK^) F_1_-*A*^*y*^/+ hybrids, respectively. Although average body length of the (B6.Cg-*A*^*y*^ × DH-Chr Y^C3H^) F_1_ hybrids was the greatest in +/+ background, the difference between (B6.Cg-*A*^*y*^ × DH-Chr Y^DH^) F_1_-+/+ and (B6.Cg-*A*^*y*^ × DH-Chr Y^C3H^) F_1_-+/+ hybrids was not statistically significant.

### Discussion

Body size is probably determined by multiple genes under the influence of non-genetic factors such as nutritional condition. To identify Y-linked gene polymorphisms associated with body size, it is essential to unify autosomal effects and to minimize non-genetic environmental influences. Thus, Y-consomic mouse strains are desirable and essential tools for investigating the effect of the Y chromosome on body size. There are several reports on the association of the Y chromosome with adult male height in humans, but the results are still contradictory [16,17].

It is a fact that many reports on body size have been obtained in human studies [18]. For example, the presence of gene associated with short stature in the pseudoautosomal region has been suggested in human [19,20]. The gene SHOX (short stature homeobox) is now considered to be involved in idiopathic growth retardation and in the short-stature phenotype of patients with Turner syndrome [18,21,22]. The pseudoautosomal localization of SHOX suggested the presence of a Y-linked functional homolog, SHOXY. However, in mice, *Shox* is not pseudoautosomal but autosomal. Therefore, the effect of the Y chromosome on body size observed in this study should not be attributed to *Shoxy*. Thus, it was suggested that there are other genes on the Y chromosome that influence body size in mice. We have genotyped Y-linked SNPs and other gene polymorphisms in these Y-consomic strains [2]. However, none of them showed polymorphisms specific to the KK/Ta strain clearly excluding these gene polymorphisms as candidates.

As a next step, it is crucial to determine at what age the body size of DH-Chr Y^KK^ strain becomes smaller than that of the other Y-consomic strains. Analysis of growth curves will be useful for this purpose. Because the difference was apparent at 80 days at the latest, the effect of the Y chromosome is expected to manifest earlier. The effect may already be apparent during the fetal period because the effect of Y chromosome on fetal growth rate has been hypothesized [23]. Comparison of birth weights will be critical to test of this hypothesis.

### Conclusion

A body-size-reducing effect of the Y chromosome of the KK/Ta mouse strain was observed irrespective of genetic background. The effect was observed in the presence of *Dh* and *A*^*y*^, the autosomal dominant mutations, both of which are known to have substantial effect on body size. These results suggest that there are Y-linked genes that control body size in mice.

## Competing interests

The author declares that he has no competing interests.

## Author’s contribution

JS designed the research, carried out experiments for data collection, analyzed the data, and wrote the manuscript.
